# Role of Serotonylation and SERT Posttranslational Modifications in Alzheimer's Disease Pathogenesis

**DOI:** 10.14336/AD.2024.0328

**Published:** 2024-03-28

**Authors:** Arubala P. Reddy, Priyanka Rawat, Nicholas Rohr, Razelle Alvir, Jasbir Bisht, Mst Anika Bushra, Jennifer Luong, Aananya P. Reddy

**Affiliations:** Nutritional Sciences Department, College of Human Sciences, Texas Tech University, Lubbock, TX 79409, USA

**Keywords:** Depression, Schizophrenia, Autism, Alzheimer's disease, posttranslational modification, Serotonin

## Abstract

The neurotransmitter serotonin (5-hydroxytryptamine, 5-HT) is implicated mainly in Alzheimer's disease (AD) and reported to be responsible for several processes and roles in the human body, such as regulating sleep, food intake, sexual behavior, anxiety, and drug abuse. It is synthesized from the amino acid tryptophan. Serotonin also functions as a signal between neurons to mature, survive, and differentiate. It plays a crucial role in neuronal plasticity, including cell migration and cell contact formation. Various psychiatric disorders, such as depression, schizophrenia, autism, and Alzheimer's disease, have been linked to an increase in serotonin-dependent signaling during the development of the nervous system. Recent studies have found 5-HT and other monoamines embedded in the nuclei of various cells, including immune cells, the peritoneal mast, and the adrenal medulla. Evidence suggests these monoamines to be involved in widespread intracellular regulation by posttranslational modifications (PTMs) of proteins. Serotonylation is the calcium-dependent process in which 5-HT forms a long-lasting covalent bond to small cytoplasmic G-proteins by endogenous transglutaminase 2 (TGM2). Serotonylation plays a role in various biological processes. The purpose of our article is to summarize historical developments and recent advances in serotonin research and serotonylation in depression, aging, AD, and other age-related neurological diseases. We also discussed several of the latest developments with Serotonin, including biological functions, pathophysiological implications and therapeutic strategies to treat patients with depression, dementia, and other age-related conditions.

## Introduction

There are numerous kinds of posttranslational modifications (PTMs), including, but not limited to, serotonylation. PTMs are enzymatic and covalent modifications that occur by adding a modifying group like methyl, phosphoryl, or acetyl to one or more amino acids of the target protein, which alters said protein's characteristics [1]. They can be reversible or irreversible. Reversible processes include covalent events, while irreversible actions consist of proteolytic alterations and are often unidirectional [1, 2]. These modifications are dynamic processes that allow for the regulation of function diversity and can alter the tertiary structure of the protein as well. The 5-HT system is arguably the most complex and expansive in the body, located in the raphe nuclei that densely innervate the whole brain [3]. It plays a significant role in mood regulation.

Classically, 5-HT is known for its role in extracellular ligand-receptor interactions but also has vital roles to play in neuronal plasticity, including cell migration and cell contact formation [3, 4]. Considering its important role in neurotransmission, close to 90-95% of the body's 5-HT is localized to the periphery [5]. For the rest of the body, 5-HT plays multiple roles in terms of one's hormones [6]. 5-HT was even identified to be the vasoconstrictor compound contained in blood serum [7]. Consequently, disruption of the 5-HT system can lead to developmental malformations, disturbances to cell division, and complete cell arrest [4]. Recent studies have found 5-HT and other monoamines embedded in the nuclei of various cells, including immune cells, the peritoneal mast, and the adrenal medulla [4]. Evidence suggests these monoamines to be involved in widespread intracellular regulation by PTMs of proteins [4, 8].

This article will cover the discovery, mechanism, and effect of Serotonin (5-HT) as a posttranslational modification. Our article also discusses recent advancements of serotonin research and serotonylation in depression, aging, AD, and other age-related neurological diseases. This article also covered several other aspects of Serotonin, including biological functions, pathophysiological implications, and therapeutic strategies to treat patients with depression, dementia, and other age-related conditions.

**Table 1:** Discovery and Overview.

Decade	Discovery	References
**1940s**	Discovery of Serotonin	[9, 10]
**1950s**	Imipramine created and 1^st^ generation antidepressant	[11-13]
**1952**	Monoamine hypothesized	[14-17]
**1957**	1957 Discovery of monoamine oxidase inhibitors (MAOIs)	[11, 18-20]
**1960s**	Development of selective serotonin reuptake inhibitors (SSRIs)	[11, 21, 22]
**1970s**	Ketamine was originally marketed as an anesthetic but proved to benefit patients with treatment-resistant depression/bipolar disorder (TRD/BPD)	[23]
**1980s**	2^nd^ generation antidepressants	[24-27]
**1990s**	Various SSRIs were tested for potency and found to be better tolerated by patients otherwise using tricyclic antidepressants (TCAs)	[28-33]
**2000s**	Research found that SSRIs are not fast-acting	[34-36]
**2010s**	Novel depression therapies continue to be sought after	[37]
**2020s**	Combined therapies have proven to yield better workarounds for discovered therapeutic limitations.	[38, 39]

**Figure 1.**
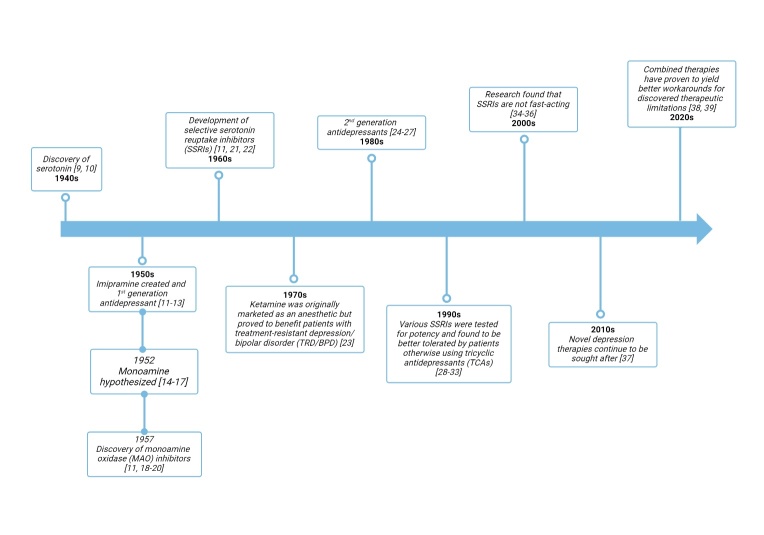
Serotonin and selective serotonin reuptake inhibitors through the years.

The intricate and complex pathology of the brainstem often called the isodendritic core (IDC), is defined by time-common motor or sensory neurons and their frequent encounter with dendritic and neuronal connections. Due to archaic anatomy, rhythmicity defines more frequent and reticular formation but not complexity at the same level. The reticular formation is characterized by attributes, territories, and distinct physiological features of noyaux ouverts- noyaux fermes (the efferent and afferent property of the IDC). The history of Serotonin and SSRIs is extensive (Refer to Fig. 1 and Table 1) The 1^st^ record of brainstem reticular formation was reported by Oliver and Boyd in 1957 and emphasized the effector connection to the cerebellum and forebrain to facilitate essential physiological functions [40]. In 1962, Hirano studied the neurofibrillary tangles (NFT) in the Chamorro population of Guam and found that amyotrophic lateral sclerosis (ALS) and Parkinsonian-like dementia complexes affected the subcortical brain and categorically showed loss of neurons in the substantial nigra (SN), dorsal raphe nucleus (DRN), and locus ceruleous (LC) [41]. He also reported the pigmentation deposition in the hippocampus and subcortical neurons for the first time [41]. The brainstem neurons were severely affected by NFT in AD, Parkinson's disease (PD) and ALS symptoms patients' brains. In 1981, MN Rossor stated the loss of IDC neurons in AD and PD but did not put much emphasis on extreme loss in presynaptic 5-HT neurons [42], further studied in 1982 when Sara Benton showed in a study of 5-HT and catecholamine (CA) uptake assay of tritiated neurotransmitters in AD and control patients. The 5-HT uptake was significantly lost in AD patients compared to control subjects [43]. It is also noted that the expected losses of neurons related to aging occur in neurotransmitter neurons, further adding to AD vulnerability in 5-HT neurons [44]. Another study in 1982 where the reuptake of radiolabeled 5-HT showed significant loss of 5-HT neurons, loss of uptake of 5-HT in the neocortex, and loss of CA activity in cholinergic neurons was reported [43]. He also reported more than Lewy body pigmentation.

The DRN neurons extend their connections to various targets across the brain, employing multiple neurotransmitters. Among these, 5-HT is the most abundant and significant [45]. Dahlstrom and Fuxe (1964) were the first to detail the structure of the serotonergic system within the DRN in rats, employing the formaldehyde-induced fluorescence (FIF) technique. This method, which allows for monoamine visualization, was initially developed by Falck et al. in 1962. Despite 5-HT being identified over 70 years ago, it's only in the past decades that Serotonin has been found to regulate physiological function through the covalent modification of substrate in a process known as serotonylation [46]. Serotonylation is the calcium-dependent process in which 5-HT forms a long-lasting covalent bond to small cytoplasmic G-proteins by endogenous TGM, such as TGM2. Other monoamine neurotransmitters include dopamine (DA), histamine (HA), and noradrenaline (NA), which can also bind to the protein's glutamine site [46]. The term "monoaminylation" has been introduced to characterize the transamidation reactions where a monoamine is added to proteins post-translationally [46]. Walther and colleagues published a review in 2011 that focused on monoaminylation, highlighting the evolutionary significance of monoamines and the enzymes responsible for their synthesis [47]. Diego Walther and colleagues found that endogenous TGM enzymes facilitate the covalent attachment of 5-HT to small guanine nucleotide-binding proteins (G proteins) in the cytoplasm, leading to the continuous activation of these small G proteins in platelets [7].

The first report of 5-HT being covalently linked to another protein was in 'COAT-platelets', where 5-HT was found to be related to procoagulant proteins on the cell surface [48]. It was found that covalently bound 5-HT residues on hyper-stimulated platelets, specifically on fibronectin, fibrinogen, von Willebrand factor, and α2 antiplasmin, acted as an adhesive to secure the proteins on the surface of the coated platelets [49]. Later studies found that the newly coined serotonylation was facilitated by TGM2 [50, 51]. Therefore, serotonylation became the first description of monoaminyl modification and the first non-methyl PTM of glutamine residues [50]. Other monoamines such as DA, NA, and the fluorescent amine mono-dansylcadaverine were found to be transamidated into fibronectin, suggesting a more general mechanism of "monoaminylation" [3, 7, 8]. However, it is still unknown if any demonoaminylases exist.

## Serotonin Synthesis Pathway and Regulation

The 5-HT is a monoamine thought to be involved in various functions in the body, such as influencing sexual behavior, regulating sleep, food intake, alcoholism, anxiety, and drug abuse. It is synthesized in a two-step process from tryptophan to produce a neurotransmitter used by the central nervous system as a whole [5]. The first limiting enzymatic step consists of hydroxylation from tryptophan hydroxylase (TPH) to form 5-hydroxytryptophan (5-HTP). Distinct activity levels between the TPH isoforms, TPH1 and TPH2, have been delineated [7].

**Figure 2.**
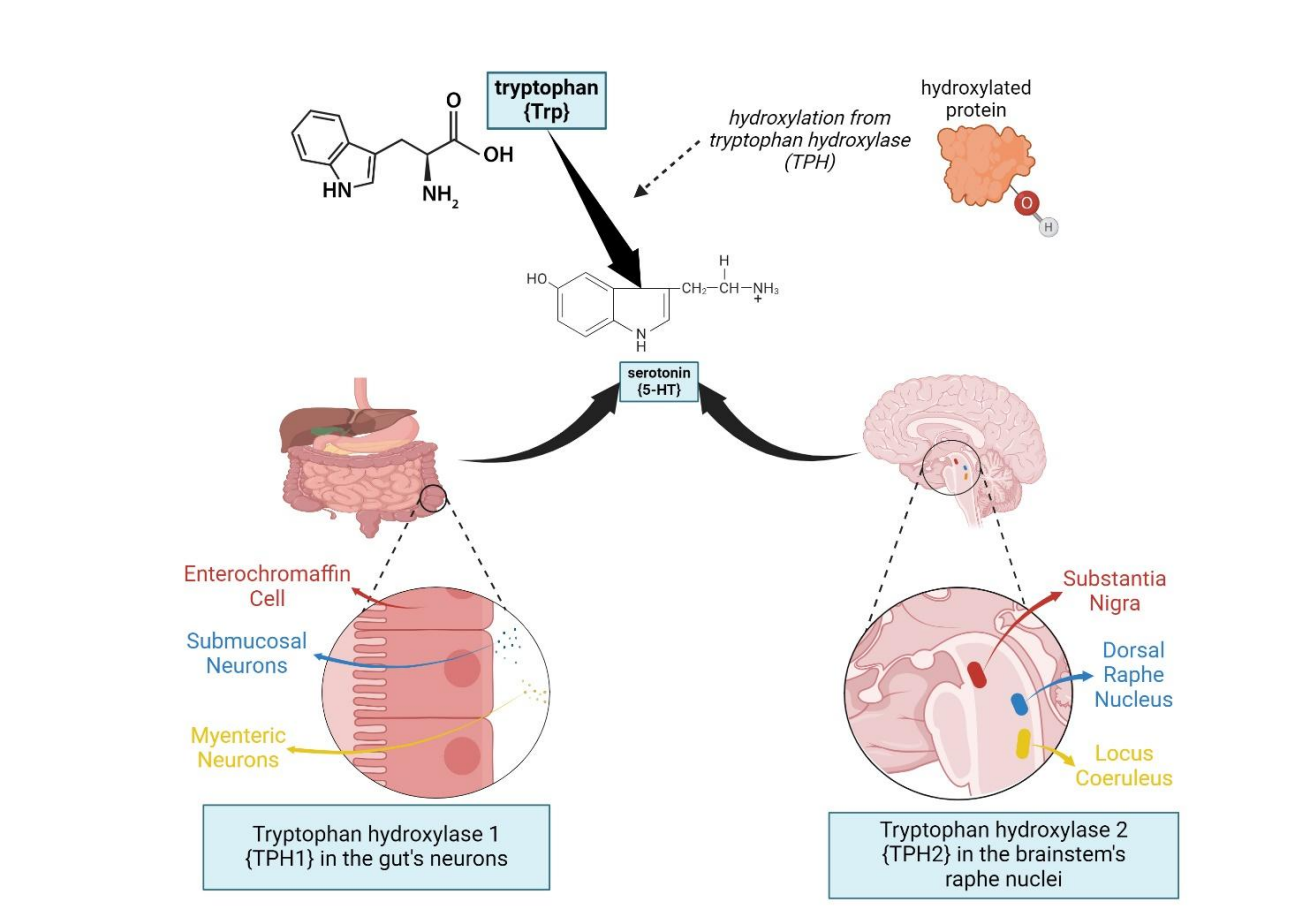
**Serotonin synthesis: TPH hydroxylates tryptophan**. TPH1 hydroxylase is in the gut's neurons while TPH2 hydroxylase is in the brainstem's raphe nuclei.

TPH1 exhibits a widespread expression in peripheral tissues, contributing to 5-HT synthesis in non-neuronal tissues. In contrast, TPH2 is selectively expressed in neurons, which governs the synthesis of 5-HT, specifically in neuronal tissues [52]. The second step is the conversion of 5-HTP to 5-HT by an aromatic L-amino acid decarboxylase. Peripheral 5-HT is derived via TPH1, which is predominantly expressed in the enterochromaffin gut cells. However, central 5-HT is derived from TPH2, which is mainly present in the brainstem raphe nuclei [53]. The activity of the enzyme TPH requires reduced pteridine cofactors such as L-erythrotetrahydrobiopterin and molecular oxygen (refer to Fig. 2).

Because 5-HT is a simple molecule, it rapidly moves into vesicles through a vesicular monoamine transporter (VMAT) after its formation [54]. Subsequently, these vesicles undergo exocytosis, releasing 5-HT into the synaptic cleft, which engages with pre- and/or postsynaptic 5-HT receptors [54]. 5-HT works locally in the gut and comes into circulation where more than 95% of 5-HT is encapsulated in platelets [55]. Circulating platelets release 5-HT in the damaged tissue, resulting in blood vessel contraction and coagulation [55]. Platelets can collect and secrete 5-HT and, therefore, modulate peripheral 5-HT levels [56]. 5-HT's effect ends when it is taken back into the cell through its transporter, which involves the sodium-dependent recycling of 5-HT [57]. Along with its receptors, 5-HT plays a crucial role as a regulator of brain function, as it is strategically positioned to influence various behavioral effects [58]. MAO, an essential mitochondrial-bound flavoenzyme, predominantly facilitates the enzymatic breakdown of 5-HT into 5-hydroxyindoleacetic acid [59, 60]. On the other hand, it can change into N-methyl, N, N-dimethyl, or O-methyl tryptamine and eventually convert into melatonin. Melatonin not only control the metabolism of beta-amyloid precursor protein and several other essential housekeeping genes within different types of cells, stimulates the initial stage of neuronal differentiation, but it also significantly reduces the level of both shorter and longer forms of toxic cortical amyloid beta, which are implicated in the Aβ plaques accumulation which is a hallmark of AD [61, 62].

## Biological Functions of Serotonin (5-HT)

**Figure 3.**
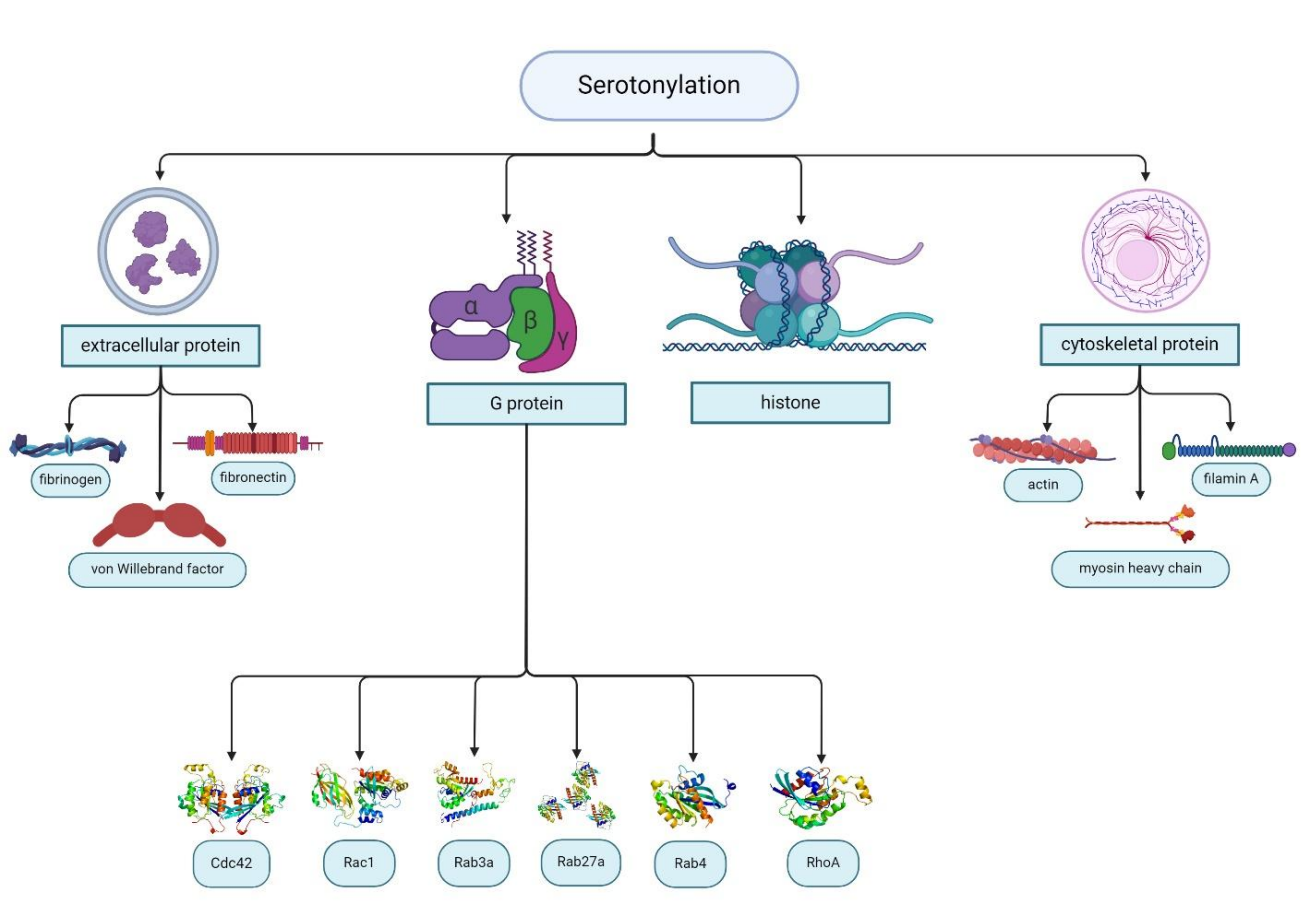
Serotonylation of different substrates is involved in various body functions.

5-HT influences various aspects of your well-being, encompassing emotions, body functions, and motor skills (refer to Fig. 3). It also plays a role in sleep, many healing processes, and digestion. Additionally, 5-HT is believed to act as a natural mood stabilizer. Beyond its function as a neurotransmitter in the central nervous system, 5-HT serves various other roles. The involvement of 5-HT in energy balance and food intake suggests signaling interactions between the gut and the brain [54]. Nevertheless, the release of 5-HT in the intestines induced by gastric distension triggers neuronal activation in the solitary tract/nucleus (brainstem) and the paraventricular nucleus of the hypothalamus. As a result, peripheral 5-HT indirectly impacts the brain [54, 63].

**Figure 4.**
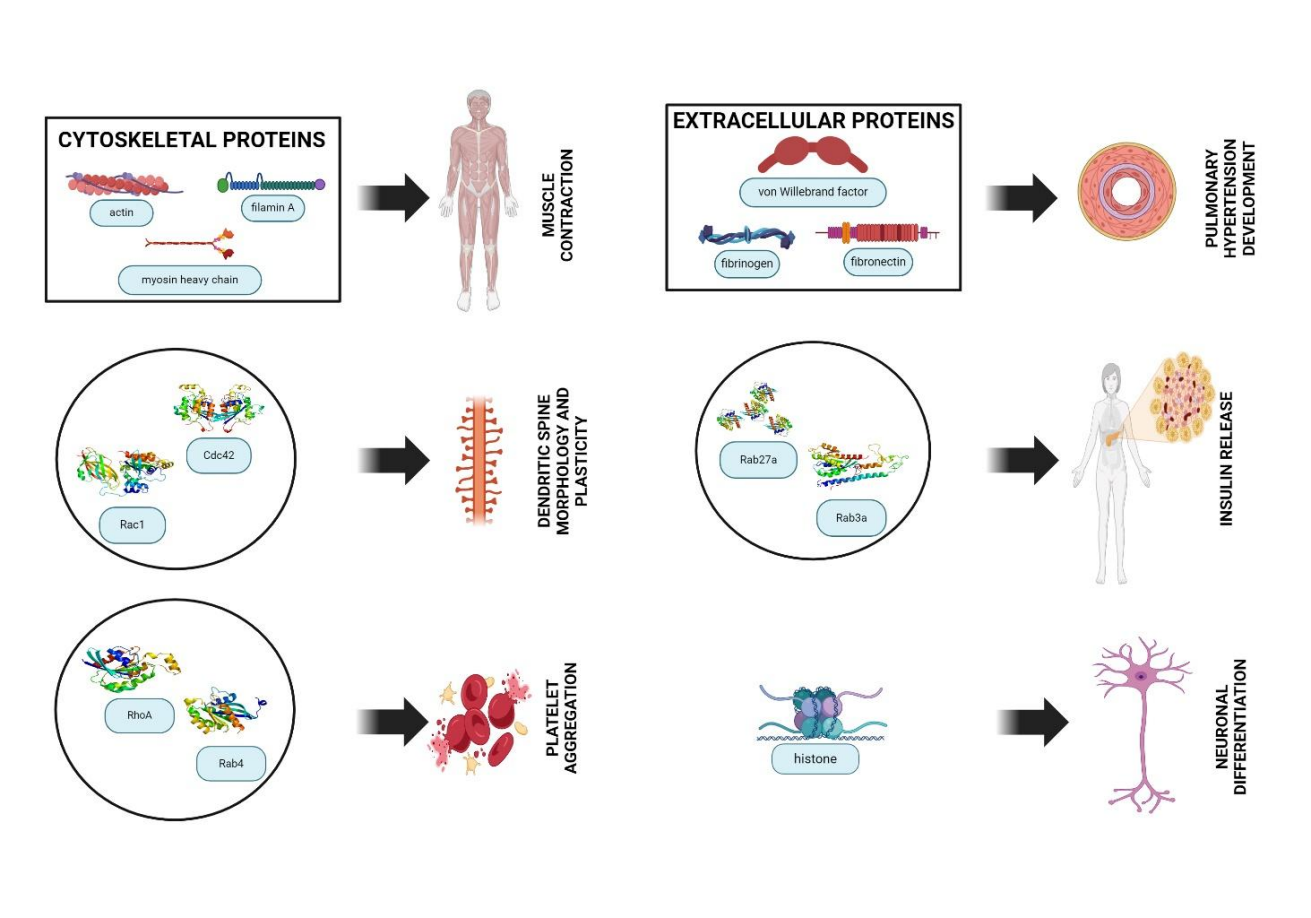
Serotonin has different substrates for serotonylation, including extracellular protein, G protein, histone, and cytoskeletal protein, and it is implicated in several physiological and pathophysiological functions.

The 5-HT plays a regulatory role in pancreatic secretion, facilitates increased insulin release, enhances glucose uptake in muscle tissue, stimulates lipogenesis in fat tissue, and contributes to lipid depositions in the liver [64] (Refer to Figure 4). 5-HT influences the central respiratory drive, elevates pulmonary vascular resistance, and can potentially induce remodeling of the pulmonary vasculature [58]. 5-HT contributes to pain perception, especially in the colon, where its effects are believed to be mediated through 5-HT4 receptors [65, 66]. 5-HT also plays a role in fluid secretion and the regulation of ion transport in both the small and large intestines across various species [67-69]. 5-HT modulates micturition, uterine muscle tone, penile detumescence, uterine vasoconstriction, and oocyte maturation [58]. 5-HT can stimulate ciliary body muscle fibers, resulting in pupil dilation and subsequent elevation of intraocular pressure [70]. 5-HT exhibits positive inotropic and chronotropic effects by augmenting intracellular calcium levels in cardiac myocytes, potentially leading to the onset of tachyarrhythmias [71]. 5-HT has the potential to induce vasodilation when the endothelium is intact and vasoconstriction when the endothelium is damaged [72].

## Pathophysiological Implications

Over the past fifty years since its identification, extensive research has been conducted on 5-HT, investigating its crucial involvement in various physiological functions such as cognition, vascular tone, insulin release, and gastrointestinal mobility. Serotonylation is also believed to play a role in multiple diseases, including diabetes, pulmonary hypertension, and thrombosis [73]. Additionally, its relevance extends to numerous disease states, encompassing depression, psychosis, migraine, and anxiety [74-77]. Consequently, in neurons of both Drosophila and mice, an artificially elevated level of 5-HT, especially in the cytoplasm, results in abnormalities reminiscent of neurodegenerative disorders [78]. Changes in 5-HT levels and signaling have been demonstrated to affect bone mass in both mice and humans [79]. With aging, the uptake of 5-HT into platelets is heightened. Consequently, the concentrations of 5-HT within the platelets increase, making it more likely for 5-HT to initiate platelet aggregation [71].

Accumulating evidence supports the involvement of 5-HT in the insulin/IGF-1 pathway and, consequently, the aging process [80]. The decrease in motor activity associated with aging in C. elegans can be quantified and subsequently restored by eliminating 5-HT receptors (ser-1 and ser-4) and instead employing 5-HT receptor antagonists [81]. Numerous investigations, including a microarray analysis in the human brain, have observed alterations in the expression of 5-HT receptors and changes in binding affinities associated with aging [82, 83]. The extensive literature indicates that 5-HT can induce ischemia by directly causing vasoconstriction in the coronary arteries of experimental animals through alterations in 5-HT2A receptors [84].

Several neuropsychological conditions, including AD and PD, clinical depression, schizophrenia, and anxiety disorders, involve a serotonergic component [83, 85]. For instance, in AD pathology, where there is degeneration of serotonergic neurons and loss of 5-HT receptors, genetic mouse models of AD reveal the presence of swollen serotonergic fibers located near amyloid plaques [78, 86]. In humans, a notable decline associated with age has been reported in the density of 5-HT transporter (SERT) in various brain regions, including the thalamus, hypothalamus, midbrain, brain stem, and diencephalon [87, 88]. Notably, a recent observational study involving septic patients in France found that sepsis did not coincide with a change in plasma 5-HT levels. Instead, there was a significant decrease in 5-HT levels within platelets (627 vs. 222 nM). This implies a potential role of 5-HT in human sepsis [89]. The viability of these findings indicates that elevated plasma 5-HT levels are associated with heart failure. For instance, a study, irrespective of age or medication, found that stable heart failure patients exhibited higher plasma 5-HT levels compared to individuals in the standard control group [90].

Hypertensive individuals have been reported to show an increased plasma concentration of 5-HT [71]. An increase in plasma 5-HT levels could potentially result in the covalent modification of the protein rab4, subsequently reducing the function of SERT in platelets. This creates a vicious cycle, as the uptake of 5-HT from the plasma into platelets diminishes, accumulating more 5-HT in the plasma [91]. All the studies mentioned above indicate the pathophysiological role of 5-HT in aging.

## Targets/Substrates for Serotonylation

Serotonylation is involved in various processes, including physiological and pathophysiological mechanisms. Serotonylation of extracellular proteins, small GTPases, histones and cytoskeletal proteins has been observed to have multiple activities in the activation of platelets, neuronal differentiation, muscle contraction, and pulmonary hypertension, which, in turn, suggests that serotonylation has significance in physiological as well as pathological roles [46, 92] (refer to Table 2 and Fig. 4).

The initial function attributed to protein serotonylation focused on extracellular proteins, and is found on hyperactivated platelets, specifically Fibrinogen, von Willebrand factor, and fibronectin [48]. These covalently bound 5-HT residues on hyperactivated platelets, with the inclusion of α2 antiplasmin, act as an adhesive to secure the proteins on the surface of these so-called coated platelets (the active form of thrombocytes) [48, 49]. Consequently, coated platelets secure an excellent number of procoagulant proteins on the surface of the cells and enable blood clot formation and activation of platelets [49, 115]. These proteins serve as acyl-donor substrates for transglutaminases and coat platelets, with fibrinogen protein being observed to undergo derivatization with 5-HT [48]. The serotonylation of fibronectin resulted in an enhanced protein accumulation in these cells' extracellular matrix [8]. Many studies found monoaminylation of fibronectin in brain tissue, which regulates the proliferation of pulmonary artery smooth muscle migration and suppresses osteoblast mineralization [3, 116, 117] (refer to Fig. 3).

## Serotonylation of Small GTPases

Serotonylation of small G proteins is one of the largest target groups of proteins, including Rac1, Rab3a, Rab4, Rab27a, and RhoA [7]. Monoaminylation leads to active GTPases, even in the GDP bound form, uncoupling nucleotide binding and hydrolysis from their activity in signaling [7]. The serotonylation of small GTPases might be responsible for the pathophysiological effect seen in pulmonary artery smooth muscle cells of enhanced SERT-mediated 5-HT transport [7].

In the synapse, small GTPase proteins such as RhoA, Rac1 and Cdc42 play a significant role in the spine's synaptic plasticity, formation, and morphogenesis [118]. Interestingly, some small G proteins, like RhoA, Rab4, Rac1 and Cdc42, are associated directly with signaling during platelet aggregation, cytoskeleton rearrangement and facilitating cytoskeleton [119-121]. A study found that the serotonylation of Rab GTPase proteins triggers these proteins in β-cells of the pancreas and enhances glucose-mediated insulin secretion [51]. The activation of GTPase proteins is connected with the translocation of GLUT4 to the cell membrane, a process that is tied to the insulin signaling pathway dependent on PI3K. Small protein Rac1 serotonylation is induced by stimulation of 5-HT2A receptors and resulting in activation of Rac1 [122]. An expansion in intracellular Ca^2+^ influx leading to the stimulation of receptors is required and enough to induce serotonylation and Rac1 activation [108]. A study found that both ligand-gated ion channels and G protein-coupled receptors can induce transamidation and Rac1 activation, and this transamidation can regulate the size of dendritic spines [99].

**Table 2:** Summary of Serotonylation of different proteins.

Proteins	Location in body	How protein is serotonylated	Correlation to disease	What protein does	References
**Rho**	Pulmonary artery	T-Gase dependent	Idiopathic Pulmonary Hypertension	Acts as a molecular switch that can bind and activate effector molecules in the GTP form	[7, 93-96]
**Fibronectin**			Pulmonary Hypertension		[73, 97, 98]
**Rab4**	Blood platelets	T-Gase dependent	• Leads to α-granule exocytosis • Platelets prone to aggregation • Increased plasma 5-HT biomarker of sudden sensorineural hearing loss	Regulates translocation of GLUT4 glucose transporter to the cell membrane in skeletal muscle cells	[7, 98-100]
**RhoA**	Smooth muscle cells, lactating mammal epithelial glands, brain/spine	Uptake of 5-HT into cells by SERT & stimulation of 5-HT2RA receptors on the cell membrane T-Gase dependent	• Idiopathic Pulmonary Hypertension • This leads to spine pruning. • Significant role in synaptic plasticity and formation and morphogenesis of the spine	After serotonylation has the potential to stimulate endocytic mechanisms or membrane ruffling	[94, 95, 101-107]
**Rab3a & Rab27a**	Pancreas within b-cells	T-Gase dependent	• Rab3a requires a rapid cycle of active and inactive cycling. • Rab27a increases insulin release and is always active	Regulates trafficking of proteins between organelles & critical for glucose-stimulated insulin secretion from pancreatic beta cells	[94, 99]
**Rac1**	Brain/spine	T-Gase dependent	• Significant role in synaptic plasticity and formation and morphogenesis of the spine • Activation promotes spine formation, growth, and stabilization.		[99, 103, 107-109]
**Cdc42**	Brain/spine		• Significant role in synaptic plasticity and formation and morphogenesis of the spine • Activation promotes spine formation, growth, and stabilization.		[103, 110, 111]
**Histone H3**	Every cell in the body	T-Gase dependent	• Patterns of K4me3 and Q5ser may strengthen patterns of inducible gene expression *in vivo*		[50, 112-114]

## Serotonylation of Histones

The chemical alteration of histones can cause various DNA-templated processes, including the process of gene transcription [123]. Histones are detailed by various PTMs, which are inducted and eliminated through chromatin-modifying enzymes, commonly known as writers and erasers [124]. These proteins diversly coordinate to specify several biological consequences [123, 125, 126]. Histone PTMs, whether individually or collectively, can modify the local function of chromatin; this alteration can occur through changes to the nucleoprotein complex's inherent structure and stability or by serving as targeting mechanisms for nuclear factors; these factors possess "reader domains" that recognize and bind to specific marks within particular sequence contexts [127]. PTMs of histone tails recruit chromatin-remodeling proteins and can make a region of the genome closed or open to transcriptional elements. Therefore, histones act as pivotal signaling hubs, playing a crucial role in regulating transcription [76]; it is not surprising that disturbances in these modifications, caused by irregular inputs or outputs, are often linked to diseases [128-130].

Histone serotonylation emerged as a novel epigenetic factor. Serotonylation is a posttranslational alteration of histones in organisms capable of 5-HT production. It entails the attachment of 5-HT molecules to histone proteins, specifically histone H3. H3 also serves as an endogenous substrate for serotonylation [50, 131]. One of the most explored epigenetic marks is the trimethylation of lysine 4 on histone H3 (H3K4me3)[132]. This evolutionary conserved PTM is associated with active transcription and is highly enriched at pro-motor regions and transcription start sites [133-135]. Recently identified histone serotonylation, particularly the modification of glutamine at position Q5 in histone H3 (H3Q5ser), is recognized as a permissive posttranslational modification that coexists with adjacent lysine 4 trimethylation H3K4me3 on the same histone tail [50].

This modification, denoted as H3K4me3Q5ser, has been identified as enhancing chromatin's affinity for transcription factors like TFIID. As a result, it activates the expression of downstream genes associated with neuronal differentiation and various brain functions [50]. While the H3K4me3Q5ser modification facilitates TFIID binding, the serotonyl mark disrupts substrate binding with KDM5B, a known oncogene, by occupying the binding pocket and creating steric clash within the catalytic center [132, 136]. Kinetic studies indicate that the activity of the H3K4 methyltransferase, MLL, is unhindered by the presence of the serotonyl group, having no impact on the installation of the first methyl group (i.e., H3K4me0→ H3K4me1) and only a very minor impact on the subsequent methylation step (i.e., H3K4me1 →H3K4me2) [132].

## Serotonylation of Cytoskeletal Proteins

Cytoskeletal proteins, like α-actin, β-actin, γ-actin, myosin heavy chain, and the actin-binding protein filamin A, constitute an additional group of targets for monoaminylation [96, 137]. It has been speculated that they are serotonylated because they are among the most abundant tissues rich in smooth muscle [96]. Of the many proteins that have been determined to get serotonylated, all contain a significant number of glutamine residues, typically between 2-3% but up to 6% in the myosin-heavy chain [96, 138]. In both rat aorta and cultured aortic smooth muscle cells (SMCs), serotonylation has been observed in critical proteins such as α-actin, β-actin, γ-actin, myosin heavy chain, and the actin-binding protein filamin A, as identified by tandem mass spectrometry [96]. Notably, the inhibition of TGM2 activity using cysteamine effectively impeded the 5-HT-induced contraction of the thoracic aorta [96]. Through mass spectrometry analysis, the serotonylation of actin has been conclusively confirmed in a colorectal cancer cell line [138]. This method allowed for the precise identification and characterization of the posttranslational modification of actin by 5-HT in the context of colorectal cancer.

## Serotonylation of Protein Kinase B (Akt)

The signaling protein Akt has been identified as a substrate for serotonylation in pulmonary arterial smooth muscle cells involving TGM2 [116]. Due to the upregulation of TGM2 in pulmonary arterial smooth muscle cells during pulmonary arterial hypertension (PAH), the serotonylation of Akt may play a role in the remodeling of pulmonary arteries in this disease [92].

## Posttranslational Modification of Serotonin Receptor

PTMs of SERT play different roles, including regulating protein folding, interaction, intracellular trafficking, and activity and degradation [139]. SERT belongs to the family of sodium- and chloride-dependent monoamine transporters, which encompasses transporters for other monoamines such as for DA and NE, in addition to transporters for creatine, proline, and γ-aminobutyric acid [93]. Several factors regulate different stages of posttranslational modifications in SERT; even the extracellular concentration of 5-HT exhibits a biphasic effect on the density of SERT molecules on the cell surface by influencing intracellular signaling and the association of SERT with other proteins [140]. SERT possesses oligomeric N-glycan structures and features disulfide bonds formed between cysteine residues within the second extracellular domains [141, 142]. Uptake activity of 5-HT by SERT depends on its cellular location and correct folding, which is controlled by various PTMs, including phosphorylation as a molecular on/off switch and ubiquitination for proteasomal degradation [48]. Many studies suggest that phosphorylation of SERT is balanced by the activity of various kinases and phosphatases, which modulate SERT expression and membrane localization. Phosphorylation on serine and threonine residues mediates the regulation of SERT [143, 144]. Interestingly, phosphorylation can affect both the conformation and activity of the SERT, and in turn, the conformational state of SERT associated with its activity can also impact its phosphorylation level [93]. It has been observed that phosphorylation plays a role in regulating both trafficking and activity of SERT [93, 145].

The SERT's glycosylation occurs within the lumen of the endoplasmic reticulum. SERT undergoes glucosylation post-translationally, where it is modified with high mannose oligosaccharide at its asparagine residues (N-glycan)[145]. N- glycosylation plays a crucial role in the ER quality control pathway, ensuring membrane proteins' proper folding and processing [146]. A study suggested that changes in the quantity or configuration of N-glycosyl groups on SERT or the formation of disulfide bonds in SERT alter its ability to associate with itself [93]. In addition, they also suggested that alteration of SERT PTMs is linked with diabetes [93].

Palmitate is reversibly attached to the sulfhydryl (SH) group of cysteine residues in S-palmitoylation through a thioester bond [139]. S-palmitoylation modulates a broad range of posttranslational activities, including protein activation localization, trafficking, interaction, and degradation, affecting several proteins differently [147]. A study showed that S-Palmitoylation of SERT promotes cell surface expression of SERT and uptake of 5-HT [139] (Refer to Fig. 3).

## Serotonylation in Alzheimer's Disease

AD is a progressive neurodegenerative disease characterized by progressive memory loss and multiple cognitive impairments. AD is neuropathologically characterized by the accumulation of amyloid beta plaques and hyperphosphorylated tau tangles in the brain [148, 149]. AD involves structural and pathological alteration in the brain throughout the disease's progression, mainly affecting the hippocampus and various cortical regions responsible for learning and memory [148].

AD and depression are significant public health concerns in the United States. Depression could serve as an early indicator of neurodegenerative disease and can be a potential risk factor for the onset of AD. Depression is a common neuropsychiatric conditions co-occurring with AD [150]. 5-HT transport (5-HTT) is found to be critical for these conditions, particularly the role of 5-HTT promoter (5-HTTP) polymorphisms [74-77].

Several studies have indicated the increased occurrence of AD among those who have depression, implying that depression may increase the risk of the neurodegenerative process [151-154]. Much evidence suggests that serotonylation of Rac1 protein can play a role in depression [155]. A study found an increase in TGM2 expression in both chronic stress-induced depressive-like behavior in mouse model of depression and in depressed suicide subjects [156]. Although there is evidence highlighting TGM2's critical role in depression, the mechanism by which stress leads to elevated TGM2 expression remains unclear. Many studies suggest that depression symptoms are a potential risk factor for AD.

Substantial evidence indicates that protein accumulating and forming inclusion bodies in neurodegenerative diseases are transmitted through TGM [157]. TGM proteins are present in neurons located in the brain areas containing inclusion bodies and within them [157]. TGMs are present in human hippocampal neurons and in AD brain colocalization with NFTs in hippocampal neurons [158]. Proteins identified as a substrate for TGM encompass the microtubule-associated tau protein (MAPT), which leads to phosphorylated tau tangles in tauopathies like AD and progressive supranuclear palsy [159-161].

The accumulation of amyloid beta peptides in AD and α-synuclein in PD is considered a spontaneous characteristic of these proteins; many studies suggest that TGase may significantly hasten the aggregation of these proteins [162-164]. Transglutaminase enzyme activity is tightly controlled and has been found to increase in brain regions susceptible to pathology in the neurodegenerative disease studied to date [157].

## Serotonin Alterations

Many studies suggest that alteration in neurotransmitters in AD/ADRD significantly regulates human behavior [165-167]. Studies have shown a loss in neurons within raphe nuclei as well as a decrease in serotonergic nerve terminals in the neocortex [165, 168, 169]. Postmortem brain studies have revealed reduced levels of 5-HT and its primary metabolite, 5-hydroxyindoleacetic acid (5-HIAA), in the CNS, especially in the temporal cortex [170-173]. Individuals with AD pathology showed a reduction in both central and peripheral 5-HT neurotransmission, as evidenced by decreased levels of 5-HT in CSF [174, 175].

Various forms of psychopathology, such as depression, suicide, aggression, anxiety, and bulimia, have been associated with reduced levels of 5-HT in the brain [176, 177].

The difference in male and female serotonin levels has also been observed. Many studies suggested a higher rate of 5-HT metabolism in the brain among females compared to males and the observation that major unipolar depression is more prevalent in women [178-180]. With age, there are alterations in the functioning of 5-HT; a decline in both the expression of multiple 5-HT receptors and their binding affinity in older adults has been observed [54, 82]. Dysfunctioning in 5-HT neurotransmission during aging has been linked to cognitive impairments [181].

The disruption of 5-HT neurotransmission in AD is consistent with the depletion of 5-HT neurons in the raphe nuclei and the resultant loss of 5-HT projection in the cortical [169, 182]. Deficits in 5-HT neurotransmission related to AD are linked to rapid cognitive decline as measured by the Mini-Mental State Examination score and to behavioral symptoms such as psychosis in addition to the overall severity of dementia [173, 183].

## Potential Strategies and Treatment

### Targeting TGM2:

TGM2 is believed to be a significant factor in the development of heart failure, inflammatory conditions, celiac disease, tissue fibrosis, cancer and neurodegenerative disease [184-187]. Strong evidence indicates that the harmful effects of TGM2 are mainly associated with its transglutaminase function, while minimal evidence points to the involvement of its GTPase activity [73].TGM2 is viewed as an ideal target for drug development, with numerous research initiatives on creating selective and potent inhibitors against TGM2 [188, 189]. Significantly, cystamine (mercaptamine) stands as the sole TGM2 inhibitor that has been commercialized and widely used for disease indications such as Huntington's chorea, mitochondrial disease, PD, Leigh disease, nephropathic cystinosis, Rett syndrome, chronic lymphocytic, leukaemia, and cystinosis [190]. Furthermore, intracellular Ca2+ ions play a crucial role in the structural transition of TGM2 into its active state, in fact chelating Ca2+ ions in the cytoplasm has proven adequate to inhibit TGM2-dependent serotonylation potentially offering a novel approach to treating disease [97, 188]. These findings suggest that targeting TGM2 might be a significant potential strategy for various chronic diseases.

### Targeting SERT:

Serotonin signaling has been linked with several diseases such as tumorigenesis and, depression and other mood disorders. SERT transports 5-HT into presynaptic neuron

Antidepressants primarily target the SERT, with initial efforts focusing on selective targeting such as selective serotonin reuptake inhibitors (SSRIs) to treat depression and other mental disorders. Many studies suggest that the SSRI citalopram has protective effects against amyloid beta and hyperphosphorylated tau which both induce dysfunction[150, 191, 192]. The recognition that achieving 80% saturation of the SET with SSRis, which act directly on the monoamine uptake transporter, is therapeutically advantageous has become widely accepted [193]. Other transporters like organic cation transporters (OCTs) and plasma membrane monoamine transporters (PMATs) also facilitate the transport of Serotonin to a lesser extent [46, 194]. The paradigm that the occupancy of SERT and its genetic variations significantly influences treatment outcome is suggested [195].

While the targeting of SERT transporters has shown to have beneficial effects in treating psychological disorders such as depression and anxiety, from a pharmacological stance, the term "selective" can be a relative term [74-77, 193, 196]. Even at therapeutic doses, targeting a 5-HT receptor subclass may have an effect on unintended targets, and as 90% of the body's 5-HT is in the periphery, this may cause disruptions to systems in the blood, heart, or gastrointestinal tract [56, 96, 196].

## Attenuation of Serotonin Biosynthesis

Intracellular serotonin acts as a precursor for protein serotonylation, and elevated serotonin levels are required for catalysis, which is dependent on TGM2 [73]. Serotonin inside the cell is used as substrate for protein serotonylation necessitating high serotonin levels for TGM2-dependent catalysis, blocking the production of Serotonin outside the brain has been identified as potential treatment for GI disorders, diabetes, obesity, fibrosis, and inflammation and concurrently, there is an increase in the development of small molecule compounds that inhibit TPH [197]. Therefore, pharmacologically blocking TPH to reduce serotonin production could be a potential treatment approach for conditions associated with serotonylation [73].

## Conclusions and Future Directions

Previous studies have taken a look into various topics in relation to serotonylation ranging from the aggregation of isoforms of TGM being more of a catch all to differences in 5-HT immunoreactivity between vertebrates and nonvertebrates to the advantages and implications of SSRIs in clinical trials as opposed to conducting cell work [3, 4, 150, 164, 191, 192]. They built upon each other and used very similar methods for immunohistochemistry and immunoblotting to achieve their study goals. Two particular studies stand out in terms of histone serotonylation. The first is Walther et al. who showed that the α-subunit of G-protein q reduces the reuptake of of VMAT2-dependent vesicles [7]. Due to such an absence, there is an accumulation of 5-HT in the cytoplasm [7]. The second is Farrelly et al. who expressed that *in vivo* states of monoaminylation may occur concurrently with serotonylation of H3 for gene expression regulation [50].

5-HT is well known for its role in cognitive processes, including both short-term and long-term memories. Most 5-HT neurons are found in the raphe nuclei, which send serotonergic projections across the brain. Research using both *in vitro* and *in vivo* models has shown that various PTMs, including serotonylation, phosphorylation, and glycosylation, impact SERT's confirmation and allow the transporters to act efficiently. An impairment in the serotonergic system has been linked to the underlying mechanism of various pathological conditions such as depression, anxiety, and other behavioral disorders. There is growing evidence that shows the role of the 5-HT system in AD and ADRD.

Transamidation appears to be applied to the fundamentals of several physiological processes. Intracellularly, it is shown to influence trafficking proteins, intracellular vesicular transport, and the dendritic spine area. The addition of a serotonyl-mark to histone proteins has been shown to correlate with euchromatin and an open transcription state, potentially developing an entirely new epigenetic factor. Extracellularly, serotonylation is essential for insulin secretion of pancreatic beta cells, platelet aggregation, and smooth muscle contraction.

SSRIs increase the concentration of 5-HT in the CSF and enhance the cognitive abilities and memory in patients with AD and ADRD. Current medication used for mental conditions like depression, diabetes, schizophrenia, and hypertension focus on targeting high affinity transporters responsible for supply monoamines required for monoaminylation and signaling molecules; therefore, monoaminylation represents a new potential therapeutic target for several disorders. Consequently, broadening the sensor spectrum for all signal molecules is crucial. Employing advanced methodologies like machine learning and computational design is instrumental in developing future genetically encoded biosensors. An added benefit of the diversifying array of biosensors is the ability to investigate the interaction and crosstalk among different monoamines and signaling molecule dynamics. This necessitates the development of biosensors with distinct spectral properties to enable simultaneous imaging of multiple signaling pathways without cross interference.

Several studies have successfully established the links between serotonylation of different proteins to both comorbidities of AD, depression and diabetes; however, much is still needed to be done. Hence, focusing on serotonylation could provide new insight into therapeutic alternatives for these conditions. Currently the techniques available for detecting serotonylation are limited, posing an obstacle to advancement in this field. The identification, visualization and purification of proteins present significant challenges due to their elusive nature. There is a critical need for more direct and efficient detection methodologies To explore understanding and characterization of protein modificaiton. Enhanced techniques would facilitate a deeper insight into the biological roles and regulatory mechanisms of serotonylation.

In conclusion, while serotonylation has been implicated in certain disease states, further extensive research is imperative to fully comprehend its implications and potential therapeutic applications.
